# Atherosclerotic cardiovascular disease in aging and the role of advanced cardiovascular imaging

**DOI:** 10.1038/s44325-024-00012-y

**Published:** 2024-08-02

**Authors:** Jie Jun Wong, Rilong Hong, Louis L. Y. Teo, Ru-San Tan, Angela S. Koh

**Affiliations:** 1 Department of Cardiology, National Heart Centre Singapore, 5 Hospital Drive, 169609 Singapore, Singapore; 2 Duke-NUS Medical School, 8 College Road, 169857 Singapore, Singapore

**Keywords:** Imaging, Inflammation, Atherosclerosis, Cardiovascular biology

## Abstract

Aging and inflammation are key drivers in the pathogenesis of cardiovascular disease. Aging is characterized by chronic, systemic, dysregulated inflammation and dysfunctional immune responses ― termed inflammaging ― that give rise to cumulative cardiovascular damage. These noxious processes promote epithelial dysfunction, immune infiltration, foam cell deposition, and calcification, which result in atherosclerotic plaque formation. With aging, epithelial and vascular smooth muscle cell senescence further contribute to atherogenesis by the acquisition of the senescence-associated secretory phenotype, consequently secreting pro-inflammatory and pro-fibrotic factors that exert autocrine and paracrine effects to perpetuate a vicious cycle of tissue aging and eventual failure. Recent evidence has affirmed the use of anti-inflammatory therapy to reduce cardiovascular risk; however, the possibility of off-target adverse effects may limit the application. Moreover, systemic inflammatory markers are not sufficiently precise in localizing cardiovascular active inflammation, and conventional cardiovascular imaging methods can only detect structural changes in late-stage disease. Targeted molecular imaging offers imaging-guided precision theragnostic and early upstream preventive approaches by delineating the cellular biological mechanisms underpinning cardiovascular inflammaging and holds the potential to revolutionize the personalized treatment of early atherosclerotic disease. Here, we examine recent developments in molecular imaging in relation to the mechanisms underlying aging-related atherosclerotic cardiovascular disease. We highlight challenges facing the translation of molecular imaging into clinical practice and propose future directions of these novel diagnostic modalities.

## Introduction

Recent evidence has reaffirmed the critical role of inflammation in the development of aging-related cardiovascular disease^1,2^. Chronic systemic smoldering inflammation, incurring damage over decades of life, results in clinical cardiovascular diseases, which represent late-stage irreversible tissue failure that often responds unsatisfactorily to conventional therapies^1,3^. Existing cardiovascular imaging methods rely heavily on detecting structural changes, usually present at advanced stages of disease. Despite knowledge of the vital role of inflammation in cardiovascular disease pathogenesis, clinically available markers of systemic inflammation are neither sufficiently precise in localizing cardiovascular-specific active inflammation nor selecting patients who may benefit from immune-modulating therapies^4–6^. Therefore, precision imaging is becoming increasingly essential as a translational tool for understanding and localizing the effects of aging and inflammation on the cardiovascular system^7^.

Atherosclerosis is a disease of aging, such that increasing age independently determines the atherosclerosis development to an extent not accounted for by traditional risk factors, such as smoking, dyslipidemia, or diabetes mellitus^8^. Premature biological aging is also associated with accelerated atherosclerosis, as atherosclerotic plaques show evidence of cellular senescence, and there is growing evidence that cellular senescence promotes atherosclerosis^9^. Much of the available evidence on molecular imaging in cardiovascular diseases is in the progression of atherosclerosis. In this Review, we examine recent developments in molecular imaging in relation to the mechanisms underlying aging-related atherosclerotic cardiovascular disease.

## Molecular advances relevant to atherosclerotic coronary artery disease in aging

Cellular aging is characterized by progressive loss of physiological integrity and increased vulnerability to cellular death, culminating in impaired tissue function^10^. Recent evidence has shown that the rate of cellular aging is at least partially predetermined by biochemical processes and genetic pathways that appear to be conserved in mammalian evolution and represent a common denominator in major human aging-related diseases, including cardiovascular disorders, metabolic disease, neurodegenerative disorders, and cancer^11–14^. Twelve interconnected hallmarks characterizing aging have been recently described: genomic instability, telomere attrition, epigenetic alterations, loss of proteostasis, disabled macroautophagy, deregulated nutrient-sensing, mitochondrial dysfunction, cellular senescence, stem cell exhaustion, altered intercellular communication, chronic inflammation, and dysbiosis^15^. Aging-related chronic inflammation occurs consequent to multiple derangements stemming from the other aging hallmarks^15^. For example, the translocation of nuclear and mitochondrial DNA into the cytosol stimulates pro-inflammatory DNA sensors and is exacerbated by ineffective autophagy^15^ Epigenetic dysregulation, deficient proteostasis, and disabled autophagy induce pro-inflammatory protein overexpression^15^. Genomic instability drives clonal hematopoiesis, leading to myeloid cell expansion bearing pro-inflammatory phenotypes^16,17^. At the same time, thymic involution diminishes the circulating T cell repertoire and favors self-antigens recognition, culminating in dysregulated immune responses and inflammation^15^. Accrual of senescent cells leads to development of a senescence-associated secretory phenotype (SASP) and release of high levels of inflammatory factors, including cytokines (e.g., interleukin (IL)−1a, IL-1b, IL6, interferon (INF)-y), chemokines (e.g., IL-8, monocyte chemoattractant protein (MCP)−2, MCP-4), proteases (e.g., matrix metalloproteinases (MMPs)), and growth regulators (e.g., soluble urokinase plasminogen activator receptor, MMPs), which promote chronic tissue inflammation and fibrosis, deplete tissue stem cell turnover, and disrupt normal protective tissue barriers^18^. SASPs, via paracrine and autocrine signaling effects, perpetuate tissue aging and progressive organ failure^19^. Aging-related loss of proteostasis, disabled autophagy, necroptosis, and release of damage-associated molecular patterns (DAMPs), free radicals, and reactive oxygen species further incite tissue inflammation, which is also exacerbated by dysregulated immune responses due to exhaustion of myeloid and lymphoid cells^20–22^.

In the vessel wall, aging drives dysregulated inflammation and is a major determinant of arterial atherosclerosis. Chronic low-grade vessel wall inflammation and cellular senescence promote endothelial dysfunction, microvascular rarefaction, and the initiation and progression of atherosclerosis, leading to thrombosis^23,24^. Vessel walls of aging arteries increased collagen, reduced elastin, higher levels of vasoconstrictive endothelin, reduced vasodilatory nitric oxide production, and increased medial layer thickness and stiffness^25^. From a young age, modified lipoproteins (e.g., low-density lipoprotein (LDL), lipoprotein (a), and other apolipoprotein (apo) B100-containing lipoproteins) begin accumulating in the vessel walls and act as DAMPs, igniting endothelial inflammation, which initiates the atherogenic process^26^. The NOD-, LRR- and pyrin domain-containing protein 3 (NLRP3) inflammasome, a pattern recognition receptor vital to the innate immune response, is a key player in low-grade chronic cardiovascular inflammation^23^. LDL cholesterol activates NLRP3 inflammasomes and induces potent cytokine release and immune cell recruitment^23,27^. Oxidized LDL (ox-LDL) further stimulates monocyte activation to produce pro-inflammatory reactive oxygen species and interleukins (e.g., IL-6)^28,29^. Concurrently, macrophages and other vascular cells take up lipoproteins and transform into lipid-laden foam cells, which form the necrotic, rupture-prone core in unstable plaques^30–32^. Foam cells, in turn, secrete cytokines and eventually undergo apoptosis, further releasing pro-inflammatory molecules, driving inflammation in a self-propagating cascade^33–35^.

The release of reactive oxygen species, including those produced during inflammation, induces endothelial dysfunction, altering endothelial permeability to lipoprotein sequestration and promoting leukocyte recruitment^29,36,37^. Concurrently, aged endothelial cells display higher NLRP3 inflammasome activity under oxidative stress^26,38^. Senescent vascular smooth muscle cells, through the release of SASP factors (e.g., MMP-9) and inflammasome component upregulation, may prim adjacent endothelial cells and other smooth muscle cells into pro-adhesive and pro-inflammatory states^19,26^. Infiltrating monocytes/macrophages further contribute to inflammation by transforming into foam cells^26^. Increased MMP expression induces collagenolysis and elastolysis, destabilizing the fibrous cap; concomitantly, the inflammatory plaque milieu drives activation of the coagulation cascade^26^. Over time, senescent cells and necrotic foam cells accumulate within the plaque core, which induces further release of pro-inflammatory molecules and various SASP factors^35^, propagating a vicious cycle of unstable plaque growth with a progressive risk of plaque rupture^26^.

Immune system aging also contributes significantly to atherogenesis. Senescent immune cells with impaired function may fail to eliminate other senescent cells, resulting in the persistence of SASP release^10^. Senescent immune cells infiltrate and accumulate in vessel walls, producing pro-inflammatory mediators and chemokines^10^. For example, TET2-deficient macrophages, associated with aging-related clonal haematopoiesis of indeterminate potential (CHIP), exhibit increased NLRP3 inflammasome-mediated interleukin-1β secretion and a greater propensity for cytokine release in response to atherogenic stimuli^26,39^; larger aortic plaques are observed in TET2-knockout animals compared to controls^39^. Ox-LDL has been shown to accelerate macrophage senescence, promote the release of pro-inflammatory cytokines, and inhibit macrophage migration^40^. Several lines of evidence suggest that the aging of immune cells other than macrophages contributes to atherosclerosis. For example, peripheral leukocyte telomere shortening, a hallmark of cellular aging, has been observed in patients with coronary artery disease and is associated with an increased risk of myocardial infarction independent of other coronary risk factors^40,41^. Increased circulating CD4^+^ and CD8^+^ T cells producing IFN-γ and expansion of senescent CD^4+^CD28^null^ T cells have been observed in patients with unstable angina compared to stable angina^42^; and increased plaque infiltration by senescent CD4^+^CD28^null^ T cells, in postmortem coronary specimens of culprit plaques compared to non-culprit lesions^43^. Senescent T cells also express perforin and granzymes, which induce surrounding epithelial cell and vascular smooth muscle cell damage^44^. Senescent CD8^+^ T cells have been observed to produce large amounts of IFN-y and tumor necrosis factor-α, which promote atherogenesis^40^. The level of peripheral senescent CD8^+^ T cells has been associated with increased mortality six months after myocardial infarction^44^. However, whether senescent T cell accumulation in either peripheral blood or atherosclerotic plaque is the cause or consequence of atherogenesis is not fully certain^44^.

## Targeted molecular approach through advanced cardiovascular imaging

Molecular imaging techniques have been applied to human and animal models to detect early atherogenesis, either directly through targeting pro-inflammatory molecules or indirectly through tracking immune cell activity, trafficking, and cell turnover.

### Targeting inflammation in vessels and plaques

Targeting specific upstream molecules involved in the inflammatory and atherogenic cascades may permit the detection of early subclinical atherosclerosis. For example, Ox-LDL vascular deposition has been successfully imaged in vivo in murine models using anti-ox-LDL-labelled magnetic resonance (MR) contrast agents, including micelles and lipid-coated ultrasmall superparamagnetic iron oxide particles (USPIO), which were later correlated on histopathology^45,46^. Ox-LDL was also successfully detected using near-infrared fluorescence-labeled monoclonal antibodies (LO1) employing fluorescence molecular tomography with micro-computed tomography in murine aortas and in ex vivo human coronary necrotic cores. The same authors developed a recombinant LO1-Fab-Cys construct fused with human CH1 and CL domains that could localize murine aortic atherosclerosis in vivo, which may potentially translate into future human clinical applications^45–47^. In this experiment, Ldlr^−/−^ mice and wild-type age-matched controls (n = 4 per group) each received either LO1-750 or IgG3-750 and MMPSense-645-FAST (MMPSense), an agent that is optically silent until cleaved by disease-related MMPs, for comparison^47^. Fluorescent molecular tomography/computed tomography (FMT/CT) at 4 hours after iv injection showed LO1-750 localization to the aorta and focal regions of the carotid and subclavian arteries, with MMPSense activation occurring in the same predefined region of interests of Ldlr^−/−^ mice. LO1-750 uptake was greater among Ldlr^−/−^ mice than wilde-type mice, and IgG3-750 accumulated at a substantially lower level than LO1-750^47^. These findings were then validated *ex-vivo* using confocal microscopy; LO1-750 was found to have localized within the arterial wall beneath endothelium and in the vicinity of MOMA-2-positive macrophages^47^.

MMP expression in early vascular inflammation has been successfully imaged using molecular tracers, including ^99m^Tc, ^123^I, ^124^I, and ^111^IN coupled tracers, capable of characterizing plaque composition and monitoring response to treatment^48,49^. For example, ^124^I-HO-MIP tracers displayed intense uptake in carotid lesions in ApoE^−/−^ mice in vivo on scintigraphy imaging, which correlated with immunohistochemistry^50^, and ^18^F-MMPi tracers demonstrated uptake in MMP-positive ApoE^−/−^ murine aortic plaques ex vivo on positron emission tomography (PET)/computed tomography (CT)^47,51^. IL-6 expression within aortic plaques was detected in vivo using MR with anti-IL-6-USPIO contrast in rabbit models that colocalized with plaque macrophage activity on histopathology^52^.

Besides targeting inflammatory molecules, advanced imaging techniques have also relied on other approaches to detect vascular inflammation. For example, ^18^F-NaF PET has been used in humans to detect in vivo arterial intimal microcalcifications associated with active inflammation and ongoing calcium deposition. ^18^F-NaF, which binds to hydroxyapatite, has shown promising results in identifying and localizing ruptured culprit and high-risk coronary plaques and abdominal aortic aneurysms and was even found to correlate with past coronary events, angina status, cardiovascular risk, and Framingham scores^7,48,53^. In a study by Joshi et al. (2014), 92 patients with acute coronary syndrome, stable angina pectoris, and carotid endarterectomy underwent ^18^F-NaF and ^18^F-FDG PET-CT, CT coronary angiography, and CT calcium scoring^53^. Maximum standard uptake values were corrected for blood pool activity in the superior vena cava to provide tissue-to-background ratios (TBRs), and focal uptake with a TBR > 25% higher than a proximal reference lesion identified ^18^F-NaF positive lesions^53^. Participants with stable angina further underwent prospective PET-CT imaging-guided radiofrequency intravascular to the ^18^F-NaF positive and negative plaques to determine the presence of microcalcifications, maximum frame necrotic core, remodeling index, and plaque classification (as thin-cap fibroatheroma, thick-cap fibroatheroma, pathological intimal thickening, or fibrocalcific plaque)^53^. Carotid endarterectomy specimens were also analyzed using *ex-vivo* PET-CT and immunohistochemistry. The investigators found in patients with acute myocardial infarction and symptomatic carotid disease, intense ^18^F-NaF uptake was localized to recent plaque rupture^53^. In patients with stable coronary artery disease, ^18^F-NaF uptake identified coronary plaques with high-risk features on intravascular ultrasound during coronary angiography, highlighting the utility of ^18^F-NaF imaging in identifying high-risk and ruptured plaques^53^.

Gallium, which permeates inflamed tissues by binding to transferrin or to local lactoferrin produced by leucocytes and shows moderate uptake in macrophage-rich LDLR^−/−^ApoB^100/100^ murine aortic plaques ex vivo after intravenous infusion^54^, has been used in ^68^Ga/^67^Ga PET to image atherosclerotic inflammation. Plaque hypoxia, a key substrate of vulnerable plaques that promotes foam-cell formation, neovascularization, and macrophage dysfunction, is a marker of vulnerable plaque progression^55^. Plaque hypoxemia has been imaged using molecular tracers targeting metabolites that accumulate during hypoxic conditions. For example, ^18^F-fluoromisonidazole in vivo uptake in carotid plaques of symptomatic patients was correlated positively with ^18^F-fluorodeoxyglucose (FDG) uptake^56^, while in vivo uptake in rabbit aortic plaques was colocalized with macrophage density and neovascularization in hypoxic regions^57^; ^18^F-EF5 administered intravenously showed uptake in hypoxic murine aortic plaques based on ex vivo histopathology using EF5 adduct-specific antibodies and pimonidazole^58^.

### Targeting immune cell activity

Macrophages are vital immune mediators in atherogenesis, and detecting increased vessel wall macrophage activity may signal inflammatory plaque activity. Several macrophage chemokine receptors have been targeted for molecular imaging to detect macrophage activity. C-C motif chemokine receptor 5 (CCR5) expression may be detected using PET/CT with ^64^Cu-DAPTA-Comb nanoparticles, which has been shown to successfully identify aortic plaques and track plaque progression and regression in vivo in ApoE^−/−^ mice^59^. The same authors found that ^64^Cu-DAPTA-Comb characterization of human carotid endarterectomy specimens displayed upregulation of CCR5 and colocalization with CD68+ human macrophages^59^. C-X-C chemokine receptor type 4 (CXCR4) expression may be detected using ^68^Ga-pentixafor. In one study, among patients with recent (within a week) stent-based reperfusion therapy for first ST-elevation myocardial infarct who underwent ^68^Ga-pentixafor PET/CT, significantly increased ^68^Ga-pentixafor uptake was seen in atherosclerotic coronary lesions, with the strongest signal in culprit lesions^60^. In another study, patients undergoing ^68^Ga-pentixafor PET/CT for other indications (e.g., interstitial lung disease, sarcoidosis, leukemia, complicated urinary infection) were found to have increased focal vessel wall uptake in the aortic tree that correlated with calcific plaque burden, maximum plaque thickness, and calcification score^61^. Lymphoma patients undergoing ^68^Ga-pentixafor PET/MRI showed increased uptake in carotid plaques that correlated with the severity of eccentric stenosis, which later colocalized with inflamed atheroma and pre-atheroma, and CXCR4 and CD68 expression on endarterectomy specimens^62^. After acute/subacute myocardial infarction, PET CT/cardiovascular magnetic resonance (CMR) ^68^Ga-pentixafor uptake in the infarcted myocardium strongly correlated with the time point of imaging with a linear decline up to day 13 after ischemia, and correlated with smaller scar volumes at follow-up (1 to 14 months later)^63^. C-C motif chemokine receptor 2 (CCR2) expression, signaling early pro-inflammatory monocyte/macrophage activity, has been imaged using ^68^Ga-DOTA-ECL1i tracers^64^. After coronary occlusion in murine models, increased left ventricular uptake of ^68^Ga-DOTA-ECL1i PET localized sites of cardiac injury in vivo that correlated inversely with echocardiographic left ventricular ejection fraction at four weeks and colocalized with ex vivo CCR2^+^ monocyte and CCR2^+^ macrophage infiltration on histopathology^64,65^.

Somatostatin receptor 2 (SSTR2), expressed by activated pro-inflammatory macrophages, have been imaged using molecular tracers (e.g., ^68^Ga-DOTA-TATE/TOC/NOC, ^111^In-DOTA-JR11), which have demonstrated uptake in inflamed, vulnerable coronary and carotid plaques in humans and in animal models^66,67^. In patients with large-vessel vasculitis, ^68^Ga-DOTATATE and ^18^F-FET-βAG-TOCA PET/MRI were used to monitor therapeutic response to IL-6 blockage with tocilizumab, including inflammation in the coronary and intracranial arteries^48,65,68^. Several tracers targeting/mitochondrial translocator protein (TSPO), highly expressed by macrophages, have been applied to detect macrophage activity and vascular inflammation. For example, ^3^H-DAA1106 and ^3^H-(R)-PK11195 have shown a good correlation with plaque macrophage content in human atherosclerotic carotid plaques in vitro using autoradiography^49,69,70^. ^11^C-PK11195 PET/CTA showed higher uptake in patients with symptomatic carotid plaques in vivo that was also associated with lower CT attenuation and was colocalized with CD68 and TSPO expressions on histopathology^71^. Accumulation of TSPO tracer, using ^18^F-GE180, was demonstrated in vivo in mice after myocardial infarction induced by coronary artery ligation, which correlated with left ventricular volumes and ejection fraction after eight weeks and colocalized with CD68^+^ macrophages infiltration ex vivo^72^. Increased TSPO ligand ^18^F-LW223 binding in the myocardial infarct territory in vivo one week after coronary artery ligation was correlated with elevated CD68^+^ macrophages ex vivo in murine models^73^, while uptake of ^18^F-PBR111 in atherosclerotic aortic plaques was preferentially colocalized to CD11b^+^ expression in ApoE^−/−^ mice^65,74^.

α7nACh receptors, expressed by immune (e.g., macrophages, T cells) and vascular (e.g., endothelial, smooth muscle) cells, function as modulators of pro-inflammatory cytokines expression and immune cell activity and have been implicated in atherogenesis^75^. Radiolabeled α7nAChR ligands, such as ^18^F-YLF-DW PET, successfully identified vulnerable carotid plaques in ApoE^−/−^ mice in vivo, which correlated with early plaque formation on immunohistochemistry ex vivo^76^. Folate receptor-β is upregulated in activated macrophages; ^99m^Tc-folate uptake on single photon emission computed tomography (SPECT) in human carotid endarterectomy specimens ex vivo has been colocalized with increased FR-β and CD163 expression ex vivo, suggesting M2-like macrophages infiltration in human carotid endarterectomy specimens^77^.

CMR using USPIO nanoparticles, taken up by plaque macrophages, was used to detect plaque inflammation in symptomatic carotid plaques in two patients in vivo, which correlated with ^18^F-FDG PET^78^. In another study, USPIO uptake in symptomatic human carotid plaques in vivo correlated with macrophage accumulation in predominantly ruptured/rupture-prone lesions on endarterectomy specimens^79^. In patients with abdominal aortic aneurysms, UPISO CMR uptake showed a modest correlation with ^18^F-FDG PET but demonstrated distinct patterns in uptake distribution, possibly explained by differences in phagocytic and glycolytic activities^80^. Dual-probe T1/T2 MRI imaging, utilizing ferumoxytol (SPIO) and a Gd3^+^ containing elastin-specific probe, detected inflammation and extracellular matrix degradation in murine abdominal aortic aneurysms in vivo and predicted fatal aneurysm rupture, demonstrating a strong correlation with macrophage accumulation and elastic fibre density on ex vivo histopathology^81^. Tri-modality imaging using magnetofluorescent ^64^Cu-DTPA nanoparticles, taken up by plaque macrophages, detected lipid- and macrophage-rich aortic and carotid atheroma using CMR and PET/CT in ApoE^−/−^ mice in vivo, which was corroborated on fluorescence reflectance imaging ex vivo^48,82^.

Of note, inflammatory cells other than macrophages, such as lymphocytes and neutrophils in inflammatory plaques, may be missed when employing monocyte/macrophage-specific tracers^83^. For example, macrophage integrin (Mac-1), a complement receptor comprising CD11b/CD18 expressed by immune cells involved in the innate response (e.g., granulocytes, myeloid-derived dendritic cells, NK cells, and activated macrophages), was found to be overexpressed in atherosclerotic plaques and infarcted myocardium^83^. Radiolabeled anti-CD11b targeting Mac-1 on SPECT/CT showed increased uptake in aortic plaques in ApoE^−/−^ mice in vivo, confirmed on ex vivo demonstration of inflammatory cell plaque infiltration^83^. Heightened CD11b-expression in circulating blood and anti-CD11b splenic uptake in ApoE^−/−^ mice were found in tandem with higher circulating lipids (e.g., triglyceride, total cholesterol, very low-density lipoprotein, LDL) and pro-inflammatory cytokines (e.g., IL-1α, IL-10, and tumour necrosis factor-α) profiles, supporting the role of CD11b^+^ immune cell recruitment in the setting of accelerated atherogenesis^83^.

Several imaging techniques detect inflammatory cell turnover as a marker of active inflammation. Active and proliferating macrophages highly take up Choline, a phospholipid component of the cellular membrane. ^18^F-choline PET uptake in symptomatic ipsilateral human carotid plaques is higher than contralateral asymptomatic plaques in vivo and correlates strongly with the degree of macrophage infiltration on endarterectomy^84^. Fluorothymidine (FLT), a thymidine analogue substrate for DNA synthesis commonly used in cancer imaging, showed increased atherosclerotic plaque uptake on ^18^F-FLT PET in ApoE^−/−^ mice, rabbits and humans, which correlated with higher proliferation of macrophages in aortic lesions and hematopoietic stem and progenitor cells in the spleen and bone marrow of murine models^85^.

### Targeting endothelial activation and immune cell migration

Adhesion proteins and receptors may also serve as molecular targets to detect immune cell migration as a marker of inflammation. Tracers targeting vascular adhesion protein 1 (VAP-1), an endothelial adhesion protein that regulates leukocyte translocation into tissues^86^, such as leukocyte ligands Siglec-10/9 conjugated to ^18^F and ^68^Ga-DOTA, have been used to detect myocardial inflammation in vivo in murine models of myocarditis and sarcoidosis, which was correlated to vascular endothelial VAP-1 expression, presence of capillary networks, and dense macrophage infiltration on histopathology^87^. ^68^Ga-DOTA-Siglec-9 PET/CT demonstrated uptake in aortic atherosclerotic plaques of LDL receptor^−/−^ApoB^100/100^ mice in vivo, correlating with VAP-1 expression on plaque endothelium and densely infiltrating macrophages, but not on non-atherosclerotic vessel wall endothelium^88^. Contrast-enhanced ultrasound (CEUS) using microbubbles containing a maleimide-thiol conjugation of anti–vascular cell adhesion molecule (VCAM)−1 nanobodies demonstrated increased uptake on early and late atherosclerotic plaques in vivo in LDL receptor and ApoB48 double-knockout mice and in ex vivo human carotid endarterectomy specimens^89^. CEUS with microbubbles bearing vWF-A1 domain has been used to detect activated endothelial vWF found in pro-thrombotic and pro-inflammatory plaques, demonstrating increasing selective signal enhancement in progressed atherosclerotic aortic plaques of ApoE^−/−^ mice in-vivo, which correlated with endothelial platelet adhesion on histology^90^. Furthermore, CEUS demonstrated therapeutic reductions in vascular endothelial inflammatory signals (e.g., P-selectin, VCAM-1, vWF) after anti-IL-1β therapy^91^.

Integrin αvβ3 receptors, signaling molecules expressed on activated endothelial cells and macrophages, have been imaged using tracers targeting arginyl-glycyl-aspartic (RGD) acid to detect plaque inflammation. ^18^F-galacto-RGD uptake on PET/CT in vivo was higher in stenotic segments compared to non-stenotic segments of human carotid plaques and was correlated with αvβ3 expression, CD68 macrophage density, and microvessel density ex vivo on endarterectomy specimens^92,93^. Increased focal ^18^F-galacto-RGD PET/CT uptake was seen in LDL receptor^−/−^ApoB^100/100^ murine aorta atherosclerotic plaques in vivo that colocalized with macrophage density and overexpression of αv and β3 integrins on histopathology^94,95^.

Liposomes are readily detected on MRI and may be a suitable vehicle for targeted MR molecular imaging^96^. For example, liposomes containing Gd3^+^ targeting α4β1 (a non-RGD binding integrin expressed on monocytes, lymphocytes, and neutrophils that binds to VCAM-1) showed in vivo uptake in inflamed aortic plaques of ApoE^−/−^ mice, which corresponded to subendothelial accumulation of monocytes/macrophages^97^.

## Non-molecular precision imaging targets of atherogenesis

CMR has been used to detect atherosclerotic plaque inflammation^48^. DCE CMR using gadolinium contrast in human carotid plaques correlated with fractional blood volume in vivo and microvessel density on endarterectomy specimens^98^. Gd-DTPA uptake in rabbit aorta in vivo positively correlated with intimal neovessels on histology^99^. However, the correlation between DCE CMR with ^18^F-FDG PET in human carotid plaques was weak, suggesting a complex relationship between varying degrees of micro-vascularization and different stages of plaque inflammation^100^. PET/MRI hybrid imaging offers simultaneous anatomical and functional plaque information and superior soft tissue contrast with the potential to identify high-risk coronary plaque characteristics, such as intraplaque hemorrhage and positive remodeling^48^. Besides the coronary arteries, CMR of the myocardium also provides information on downstream myocardial injury, perfusion deficit, and extent of myocardial ischemia and inflammation^101^.

CEUS techniques have been used to characterize atherosclerotic plaques. For example, the periadventitial contrast signal on CEUS using microbubbles was able to localize human carotid plaques in vivo, which correlated with intima-media thickness^102^. CEUS, using perflutren lipid microspheres, could characterize aortic plaque neovascularization in rabbit aortas based on in vivo echogenicity, which was correlated with neovessel and macrophage densities on histopathology^103^. Superb microvascular imaging, a non-invasive technique that removes noise while preserving low-velocity flow signal without requiring contrast, has been shown to detect carotid intraplaque neovascularization accurately in symptomatic patients, which was correlated with higher peak intensity on CEUS and with increased areas of inflammation on endarterectomy^104^.

Other imaging techniques to measure coronary inflammation and inflammatory risk include fat attenuation index (FAI) and epicardial adipose tissue (EAT) assessment. Perivascular FAI on CT imaging measures peri-coronary adipocyte size and lipid content. Inflammation reduces lipid accumulation and slows preadipocyte differentiation^105^. Plaques with high FAI values exhibited more vulnerable plaque characteristics (e.g., spotty calcification, low attenuation) and were associated with a pro-inflammatory coronary blood cytokine profile^106^. Epicardial adiposity has been implicated in cardiovascular pathogenesis, including coronary artery disease, heart failure, and atrial fibrillation, through several mechanisms, including paracrine and vasocrine secretion of pro-inflammatory cytokines and pro-atherogenic molecules and glucose and lipid metabolism^107^. In one meta-analysis, EAT thickness and volume of adult patients measured on CT and echocardiography were associated with high risks of myocardial infarction, coronary revascularization, and major adverse cardiovascular events^108^. Therefore, quantifying peri-coronary FAI and epicardial adiposity may be valuable markers of early vascular inflammation and atherosclerotic risks.

## Future directions

Molecular imaging holds great promise in detecting the inflammation that underlies aging-related atherogenesis while offering precision theragnostics and early upstream preventive approaches to preclinical disease. Targeted molecular imaging provides insights into the biological cellular mechanisms driving specific aging-related cardiovascular diseases. It offers a therapeutic window to deliver precision senotherapeutics and anti-inflammatory treatments before the onset of established calcific atherosclerosis.

Undoubtedly, advanced cardiovascular imaging has the potential to revolutionize atherosclerotic cardiovascular disease towards more precise and upstream approaches. Integrated strategies that account for individual susceptibility and novel and traditional risk factors, including omics, would refine the path toward precision cardiology in atherosclerotic cardiovascular disease. As aging is characterized by chronic systemic inflammation, targeting early upstream cardiovascular inflammation can potentially prevent the onset of established calcific atherosclerosis. However, most existing literature on molecular cardiovascular imaging techniques has focused on detecting early atherosclerotic inflammation; there is still vast research potential for future precision imaging modalities that target other hallmarks of aging, especially in non-atherosclerotic aging-related cardiovascular diseases such as atrial fibrillation, heart failure, and aortic valve stenosis^109,110^.

Despite the promising results, the horizon for precision imaging is still fraught with challenges. It will warrant more human research and real-world data, including readiness and navigating the varied priorities of diverse healthcare systems. There is a dire need for more real-world data from population-based cohort studies that have incorporated large-scale cohort imaging, such as the MESA Study (Multi-Ethnic Study of Atherosclerosis), as well as cohorts and clinical trials that have pre-specified aging as a risk factor for cardiovascular before disease diagnosis^111,112^. In the absence of real-world data to guide proper trials, advanced imaging remains an approach that is confined to research laboratories.

Another major limitation is the lack of currently available human research data in molecular imaging. Radiolabelled molecular probes can exert drug-like effects, and toxicity must be considered. Strict regulatory standards and safety clinical trials are required before molecular probes can be used in human research. Cost is another critical issue facing molecular imaging, including the costs of imaging machines, materials to produce imaging agents, and clinical trials. These practical issues must be addressed before molecular imaging can be safely and effectively implemented in clinical practice.

Human research is critical before the clinical implementation of molecular imaging can be achieved. Fundamentally, human arteries differ from animal arteries, and research using animal models may not apply. For example, in mice, the arterial intima consists of only endothelium overlying the internal elastic lamina, lacking smooth muscle cells or connective tissues found in humans^113^; the tunica media is also less thick and the vasa vasorium is absent, which explains the absence of ingrowth neovessels into atherosclerotic lesions^114^. Thus, in mice, monocyte recruitment in the early stages of development must predominate to form the intimal layer^115,116^. In humans, proliferation markers expressed by cells in the intimal layer and the proliferation of resident macrophages may contribute to the early inflammatory response, leading to the expansion of early lesions in humans but not mice^117^. There is also controversy about the role of macrophage scavenger receptor (MSR-1) expression in atherogenesis and its effect on lesion composition and area, which appears to differ in different mouse models^118^. Given the limitations of animal models, research in humans is a critical step to undertake before precision molecular imaging can be adopted in humans.

Another major challenge in targeting senescence/inflammation will be differentiating the physiological versus pathological effects of senescence. Normal senescence and inflammation play important physiological roles in maintaining tissue homeostasis^119^, remodeling, repair^120^, wound healing^121^, and limiting tumor cell transformation^122^. In this context, senescence is an endogenous pro-resolving mechanism that, when dysregulated or when senescent cells are not cleared efficiently, may lead to sustained inflammation or damage^123^. Therefore, determining the pathological level of senescence/inflammation or the threshold for intervention is the next critical, pertinent question for targeting cellular senescence^123^.
